# Milk fermentation products of *L. helveticus *R389 activate calcineurin as a signal to promote gut mucosal immunity

**DOI:** 10.1186/1471-2172-8-19

**Published:** 2007-09-07

**Authors:** Gabriel Vinderola, Chantal Matar, Gabriela Perdigón

**Affiliations:** 1 Centro de Referencia para Lactobacilos (CERELA-CONICET), Chacabuco 145, Tucumán (4000), Argentina; 2 Cátedra de Inmunología, Instituto de Microbiología, Facultad de Bioquímica, Química y Farmacia, Universidad Nacional de Tucumán, Tucumán, Argentina; 3 Département de Chimie et Biochimie, Université de Moncton, Moncton (NB) E1A 3E9, Canada

## Abstract

**Background:**

Fermented milks containing probiotic bacteria are a way of delivering bioactive constituents to targets in the gastrointestinal tract. We reported previously that the fermentation of milk at constant pH 6 by *L. helveticus *R389 increased its content of peptide fractions, and the oral administration of the non-bacterial fraction (FMSpH6) to mice increased total secretory IgA in the intestinal lumen and enhanced the number of IgA and various cytokines producing cells as well as the secretion of IL-6 by small intestine epithelial cells. We also demonstrated that this FMSpH6 was effective for the prevention of *Salmonella typhimurium *infection in mice. In this work, we studied in mice the impact of the oral administration of the supernatant of milk fermented by *L. helveticus *R389 on the gut physiology by measuring parameters such as calcium channels and E-cadherin expression, the activation of the biological signal calcineurin and mast and goblet cells, as a way to determine some mechanisms involved in the immunomodulating effects of the milk fermentation products, observed in previous studies. We analyzed the impact of the supernatant of milk fermented by *L. helveticus *R389 at pH6-controlled on the expression of calcineurin and on the reinforcement of the ephitelial barrier, measuring parameters such as calcium channels and E-cadherin expression and in the reinforcement of the non-specific immunity determining mast cells and goblet cells associated to the gut.

**Results:**

We observed an enhanced expression of TRPV6 channels in the duodenum, indicating an improved capacity for dietary Ca2+ uptake. We demonstrated an enhanced expression of calcineurin in the small intestine, able to upregulate immune parameters such as IL-2 and TNF production, with an increase in the number of these cytokines secreting cells. We determined an increase in the number of mucosal mast cells and goblet cells, which would mean an improved state of mucosal surveillance at sites of infection.

**Conclusion:**

The oral administration of the supernatant of milk fermented by *L. helveticus *R389 enhanced the gut mucosal immunity by improving the mechanisms that reinforce the epithelial and non-specific barriers and the gut functioning at sites of infection, with an improvement in the expression of the enzyme calcineurin, an important signal in the network that activates the gut immune system. The results of this work contribute to revealing the mechanisms underlying the immunomodulation of the gut immune function by fermented milks with probiotic bacteria.

## Background

Fermented milks with probiotic microorganisms are a way of delivering active constituents to targets in the gastrointestinal tract. Advances in probiotic development include the knowledge of the active constituents responsible for each effect, of the target sites and of the pharmacokinetics of probiotics. Most pharmacokinetic studies have described the fate of probiotics (i.e. usually their 'survival') in the gastrointestinal tract. In fact, the active constituents are seldom known, and their pharmacokinetics, except for those of lactase from yogurt bacteria, cannot therefore be assessed [1].

Milk is recognized as a nutritional source of high quality proteins. Although milk proteins have long been considered an important source of amino acids, milk proteins have also the potential to produce biologically active peptides. Various bioactive peptides have been isolated from hydrolysates of casein, which including opioid agonists or antagonists, angiotensin-converting enzyme inhibitors, and immunostimulating peptides [2,3]. Peptides derived from milk fermentation appear to survive gastrointestinal digestion and have been recovered from faeces; therefore, it seems probable that the peptides generated by the bacteria found in yogurt or other fermented milks affect gut and immune cells directly [3]. Additionally, compounds other than peptides may also be produced by bacterial fermentation [4]. Among lactic acid bacteria, *Lactobacillus helveticus *is known to possess elevated amounts of proteolytic activity [5,6], which maximizes the probabilities of releasing bioactive peptide [7]. Laffineur *et al*. [8] reported that supernatants of milk fermented by *L. helveticus *were able to modulate the *in vitro *proliferation of lymphocytes by acting on cytokine production. Matar *et al*. [9], using the proteolytic strain *L. helveticus *R389, demonstrated that the bioactive compounds released during milk fermentation possessed inmunoenhancing and antimutagenic properties not present in the non-proteolytic mutant. LeBlanc *et al*. [10] demonstrated that the peptide fractions released from milk proteins by the same strain were able to induce antitumour activity. Additionally, the protective humoral immune response after an *Escherichia coli *O157:H7 infection in mice was also demonstrated for these peptidic fractions [11]. Working with non-bacterial fractions of the fermented milk kefir, we demonstrated that the cell-free extracts were able to induce the *in vivo *production of cytokines by peritoneal macrophages and adherent cells isolated from Peyer's patches [12] and the proliferation of IgA- and cytokine-producing cells in the gut mucosa [13]. In a previous work [14] we reported that the fermentation of pH 6-controlled milk by *L. helveticus *R389 maximized the content of peptide fractions in the product. We also demonstrated that the non-bacterial fraction of this fermented milk was able to induce the proliferation of IgA+ B cells, and the increase in the number of IL-2+, IL-6+, IL-10+, TNFα+ and IFNγ+ producing cells, the secretion of IL-6 by small intestine epithelial cells and the luminal content of natural secretory IgA (S-IgA), without synthesis of antibodies directed against this fraction. We also determined the effectiveness of this non-bacterial fraction in the prevention against *S. typhimurim *infection [15]. In this work, we aimed at further characterizing a biological signal involved in the immunomodulating effects observed for this non-bacterial fraction, obtained from milk fermented by *L. helveticus *R 389. We analyzed whether this soluble fraction increased the expression of the enzyme calcineurin involved in the regulation of IL2 and TNFα cytokines production. Calcineurin is a Ca++-dependant enzyme able to regulate the production of IL-2 and TNFα [16]. Its expression would play a key role in initiating the innate immune response due to the capacity of the cytokine TNFα of initiating the cross-talk among immune cells [17]. The activation of calcineurin is also involved in key aspects of the adaptive immune response since calcineurin mediates IL-2 production, which links the innate and adaptive responses [18]. Thus we studied the impact of this supernatant on gut physiology using parameters such as the expression of calcium channels and E-cadherin and the effect of this fermented milk supernatant on mast cells and goblet cells associated with the gut, which are able to produce substances involved in intestinal pathogens clearance or mucus, respectively, both important to reinforce the non-specific intestinal barrier.

## Methods

### Animals and bacterial strains

Six-to-eight week-old BALB/c mice weighing from 20 to 25 g were obtained from the random bred colony kept by our department at CERELA. Each experimental group consisted of 5 mice housed in metallic cages kept in a controlled atmosphere (temperature 22 ± 2°C; humidity 55 ± 2%) with a 12 h light/dark cycle. Protocols involving the use of animals were approved by the Animals Protection Committee of the Research Centre CERELA.

The strain *Lactobacillus helveticus *R389, isolated from Swiss cheese, was used for this study. Overnight cultures (16 h, 37°C, aerobic conditions) were obtained in 12% skim milk (Difco, Becton Dickson and Company, Sparks, MD, USA) or in MRS broth (Britania, Buenos Aires, Argentina).

### Production of fermented milks (without pH control and pH 6-controlled) and their non-bacterial fractions

Two types of fermented milks were produced with *L. helveticus *R389: a) a fermented milk (without pH control) obtained by the inoculation (2%) (approximately 10^6 ^CFU/ml) of *L. helveticus *R389 in 12% skim milk and overnight incubation (16 h 37°C aerobiosis) and b) at pH 6-controlled fermented milk. To obtain the latter, 12% (w/v) skim milk (1 l) was inoculated (2%) (approximately 10^6 ^CFU/ml) with an overnight culture (16 h) of *L. helveticus *R389 and it was fermented using a Bioflo Model 70 13 70 biofermentor (New Brunswick Scientific) at 37°C with an agitation rate of 100 rpm and CO_2 _spurging (10 lb/in^2^; 0.2 l/min). The pH was buffered to 6.00 by the automatic addition of 8 M NaOH as required during the fermentation.

In order to obtain the non-bacterial fractions of these fermented milks, the milk obtained by overnight fermentation (16 h, without pH control, final pH 3.7) was centrifuged (3500 × g, 4°C 15 min, IEC B-22M centrifuge) and the supernatant was recovered and kept frozen (-80°C) until use (from now on referred to as fermented milk supernatant (FMS). For pH 6-controlled fermented milk, pH was lowered to 3.7 by the addition of 85% DL-lactic acid syrup (Sigma-Aldrich, St. Louis, MO, USA) and it was then centrifuged in the same conditions as previously described. The supernatant was recovered, adjusted to pH 6 and kept frozen (-80°C) until use (from now on referred to as FMSpH6). The total protein content of the non-bacterial fractions was determined by the Bradford technique (Quick Start Bradford Protein Assay, Bio-Rad Laboratories, Hercules, CA, USA). We tested only the protein content in the supernatant of the fermented milk, to performed the study in similar conditions as in the previous papers [14,15].

### Determination of calcineurin expression after samples administration

For the study of calcineurin expression in the lamina propria of the small intestine, we determined first the time-course expression of calcineurin only when FMSpH6 was administered. Groups of mice received by gavage a single dose of the FMSpH6, containing 100 μg of total protein. Control animals received phosphate buffered saline (PBS, pH 7.4) solution instead. At various times (5 min, 15 min, 30 min, 1 h, 2 h or 3 h) after the administration of the FMSpH6, animals were anesthetized and sacrificed by cervical dislocation. The small intestine was removed for histological preparation following Sainte-Marie's technique [19]. After determining the optimal time for calcineurin expression, groups of mice received a single dose (0.2 ml) of 1) FMSpH6, 2) milk fermented with *L. helveticus *R389 (10^8 ^CFU/ml) (overnight culture (16 h), without pH control), 3) 100 μl of a suspension of viable cells of *L. helveticus *R389 (10^8 ^CFU/ml) obtained in MRS broth, centrifuged (10 min, 4°C 5000 × g) and washed twice with PBS, or 4) 0,2 ml PBS (control animals). Animals were anesthetized and sacrificed by cervical dislocation 15 or 30 min (optimal time for calcineurin expression previously determined) after the administration of each sample. The small intestine was removed for histological preparation following Sainte-Marie's technique [19].

### Immunofluorescence tests for the study of the expression of calcineurin

Histological slices from the small intestine of treated or control animals were deparaffinized and rehydrated in a graded ethanol series and then incubated for 30 min in a 1% blocking solution of BSA (Jackson Immuno Research, West Grove, PA, USA) at room temperature. Histological slices were then incubated for 16 h at 4°C with monoclonal anti-calcineurin (BD Biosciences Pharmingen, San Diego, CA, USA). The incubation was followed by two washes with PBS solution and, finally, sections were treated for 45 min at 37°C with a dilution of a goat anti-mouse antibody conjugated with FITC (Jackson Immuno Research). The results were expressed as the number of calcineurin-expressing cells (positive: fluorescent cell) per 10 fields (magnification 1000×). Data represent the mean of three histological slices for each animal for each period assayed.

### Immunofluorescence tests for the study of the expression of E-cadherin and TRPV6 calcium channel, IL-2+ and TNF+ cells, CD4+ and CD8+ T lymphocytes

In order to analyze the biological impact of the increased expression of calcineurin, we studied the number of IL2 and TNFα cytokine producing cells and the number of CD4+ and CD8+ T lymphocytes in the lamina propria of the small intestine. The expression of E-cadherin and TRPV6 calcium channels was evidenced in the brush borders of ephitelial cells of the villi and in the duodenum respectively, in animals that received by gavage a single daily dose of 0.2 ml (100 μg of protein) of the FMSpH6 for 2, 5 or 7 consecutive days. Control animals received 0.2 ml PBS instead in the same way as the treated mice. All animals received, simultaneously and *ad libitum*, a sterile conventional balanced diet (proteins 230 g/kg, raw fiber 60 g/kg, total minerals 100 g/kg, Ca 13 g/kg, P 8 g/kg, moisture and vitamins 120 g/kg). Treated and untreated mice were sacrificed on the same day (2, 5 or 7).

The small intestine was removed on days 2, 5 or 7 for histological preparation following Sainte-Marie's technique [19]. Duodenum was separated only for 7 days of administration of FMS pH6 and processed in the same way for calcium channel determination by immunofluorescence assay using the TRPV6 antibody (BD Biosciences Pharmingen, San Diego, CA, USA).

IL-2+ and TNF-α+ cells were studied by an indirect immunofluorescence method. Histological slices were deparaffinized and rehydrated in a graded ethanol series and then incubated for 30 min in a 1% blocking solution of BSA (Jackson Immuno Research, West Grove, PA, USA) at room temperature. Histological slices were then incubated for 60 min at 37°C with rabbit anti-mouse IL-2 or TNF-α (Peprotech, Inc., Rocky Hill, NJ, USA) polyclonal antibodies diluted 1:100 in PBS containing 0.1% Saponin (Sigma). The incubation was followed by two washes with PBS-Saponin solution. Finally, sections were incubated for 45 min at 37°C with a dilution (1:100) of goat anti-rabbit antibody conjugated with FITC (Jackson Immuno Research) in PBS-Saponin. The incubation was followed by two washes with PBS-Saponin solution. Results were expressed as the number of positive+ (fluorescent) cells/10 fields.

The determination of CD4+ and CD8+ T lymphocytes was performed in the small intestine lamina propria of the animals given PBS (control) and FMS pH6 for 2, 5 or 7 consecutive days. CD4+ cells were determined using FITC anti-mouse CD4 (L3/T4) monoclonal antibody and FITC anti-mouse and for CD8a (Ly2) monoclonal antibody (Cederlane Laboratories, Ont., Canada) was used. The results were expressed as the number of X-producing cells (positive: fluorescent cell) per 10 fields (magnification 1000×). Data represent the mean of three histological slices for each animal for each feeding period.

For the study of TRPV6 calcium channel expression, histological slices from duodenum were treated with affinity purified goat anti-mouse TRPV6 (E-16) polyclonal antibody (Santa Cruz Biotechnology, California, USA). As secondary antibodies, goat anti-rabbit antibody conjugated with FITC or FITC conjugated affinity pure rabbit anti-goat antibody (Jackson Immuno Research) were used, respectively. For the qualitative study of E-cadherin, anti-pan cadherin developed in rabbit (Sigma Aldrich, St. Louis, MO, USA). In both assays the presence of immunofluorescence was observed.

### Histological examination of the small intestine for mast and goblet cells determination

Serial paraffin sections (4 μm) of small intestine from animals treated for 2, 5 or 7 days with FMSpH6 and from PBS control animals were stained with haematoxilin-eosin followed by light microscopy examination. Mucosal mast cells were studied by selective staining of granule content with Alcian Blue/Safranin. Goblet cells stained blue with this methodology. Briefly, histological slices were deparaffinized and rehydrated in a graded ethanol series and then incubated (room temperature) for 150 min in 1% Alcian Blue 8Gx solution in 3% acetic acid. Histological slices were then incubated for 6 min in an eosin solution and for 40 min in 0.5% safranin solution in 0.1 N HCl. Histological slices were then dehydrated in a series of ethanol and finally mounted using Canada balsam synthetic (Biopack, Buenos Aires, Argentina). The results were expressed as the number of goblet or mast cells per 10 fields (magnification 1000×). Data represent the mean of three histological slices for each animal for each feeding period.

### Statistical Analysis

Data were analyzed using the one-way ANOVA procedure of SPSS software. The differences between means were detected by the Duncan's Multiple Range Test [20]. Data were considered significantly different when *P *< 0.05.

## Results

### Determination of time course expression of calcineurin and the effect of FMSpH6 in the number of cells expressing calcineurin

**Figure 1:**
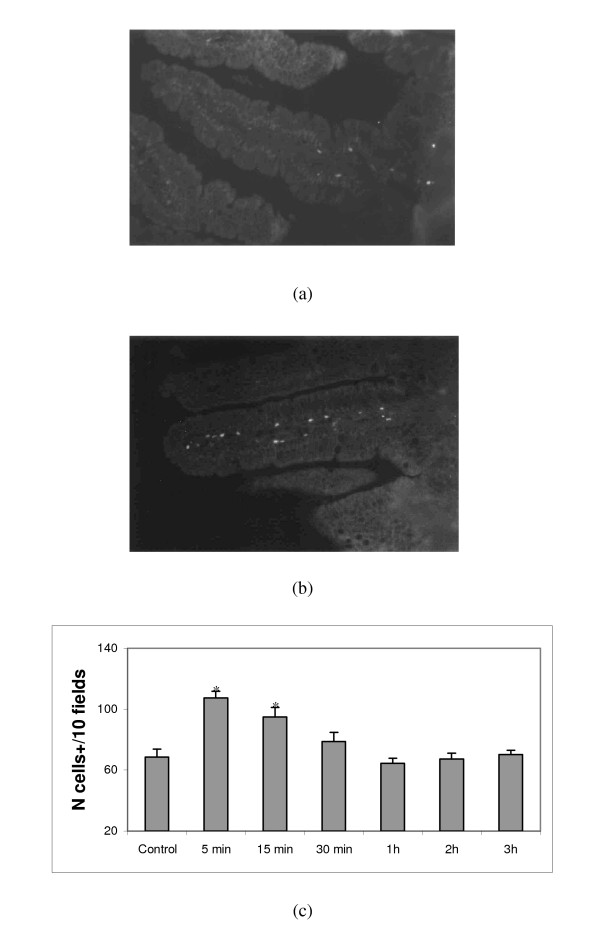
**Time course of calcineurin expression in the small intestine**. Calcineurin+ cells in the small intestine lamina propria of PBS control mice (a) and 15 min after receiving (by gavage) the FMSph6 (b). Magnification 400×. Number of calcineurin+ cells in the small intestine lamina propria of mice that received the FMSpH6 compared to PBS control animals (c). * Significantly different (*P *< 0.05) from control mice.

An antibody directed against calcineurin was used to study the time-course expression of this enzyme in the intestine following the administration of the fermented milk supernatant of milk fermented with *L. helveticus *R389 at pH 6-controlled (referred to as FMSpH6). Pictures in Figure 1 show histological slices of villi from the small intestine of control (a) and treated mice (b). Within the first 15 min of the oral administration of the FMSpH6, an enhanced expression of calcineurin+ cells was observed (Fig. 1c) that returned to control values one hour later. The changes in the number of calcineurin+ cells occurred within the first 30 min of the oral administration of the FMSpH6.

**Figure 2:**
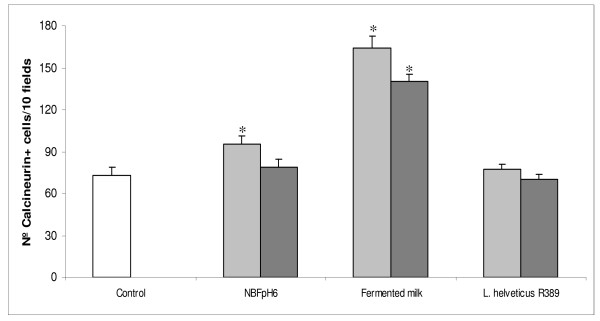
**Calcineurin expression in the small intestine after different treatments**. Number of calcineurin+ cells in the small intestine lamina propria of control mice and treated animals 15 (grey bar) or 30 (black bar) min after receiving the FMSpH6, milk fermented by *L. helveticus *R389, *L. helveticus *R389 (a suspension of viable cells) or PBS (control). * Significantly different (*P *< 0.05) from control mice.

Figure 2 shows that when mice received the milk fermented with *L. helveticus *R389 an enhance in the number of calcineurin+ cells of greater magnitude that when mice received the FMSpH6 was observed while a pure culture of *L. helveticus *R389 failed to enhance calcineurin expression.

### Study of the effect of FMSpH6 on the expression of calcium channel and E -cadherin

**Figure 3:**
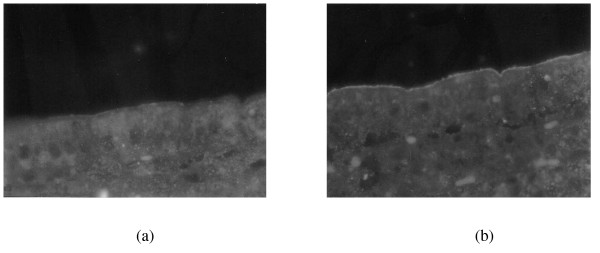
**Effect of FMSpH6 administration on TRPV6 calcium channels expression**. Effects of the oral administration of the FMSpH6 for 7 days on the expression of TRPV6 calcium channels in PBS control (a) and treated animals (b). Magnification 1000×.

The immunofluorescence examination for the expression of the calcium channel TRPV6 in the proximal intestine of mice showed immunopositive staining for TRPV6 in the brush-border membranes of the ephitelial duodenum in comparison with the control (Fig. 3a). Absorptive epithelial cells in the duodenum stained intensely for TRPV6 for FMSpH6-treated animals (Fig. 3b) whereas crypt cells were negative. The expression of E-cadherin in the brush-border membranes of the villi was not modified compared to control animals (pictures not shown).

### Histological studies of the small intestine, effect on T cell population and IL2 and TNFα cytokine positive cells induced by FMSpH6

**Figure 4:**
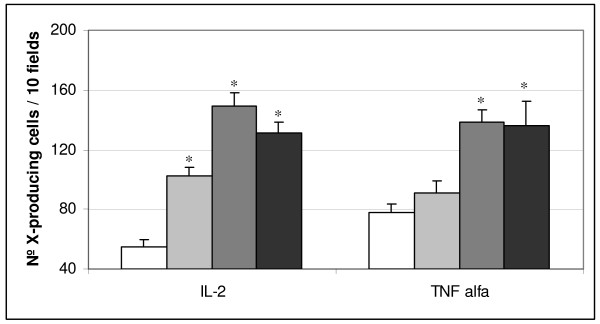
**Effect of FMSpH6 administration on IL-2+ andTNFα+ expression**. Effects of the oral administration of 12% skim milk or FMSpH6 for 2 (grey bar), 5 (grey bar) or 7 (black bar) consecutive days on the number of IL-2+ and TNFα+ cells in the small intestine lamina propria of mice compared to PBS control animals (control). * Significantly different (*P *< 0.05) from control mice.

The histological examination of slices from the small intestine of mice that received the FMSpH6 for 2, 5 or 7 consecutive days showed a slight lymphocyte infiltration in the lamina propria compared to PBS control mice. The presence of edema or mucosal atrophy was not observed (pictures not shown). When we analyzed cytokine production (Fig. 4), we observed that the oral administration of the FMSpH6 increased the number of IL-2+ or TNFα+ cells for all feeding periods assayed, compared to PBS control treatment. There was a significant increase in the number of CD4+ cells when animals received the FMSpH6 for 7 consecutive days (43 ± 3 cells/10 fields), compared to control animals (32 ± 4 cells/10 fields), while there was no effect on the CD8 T lymphocyte population compared to the PBS control (26 ± 1 cells/10 fields).

### Effect of FMSpH6 on the number of goblet and mast cells

**Figure 5:**
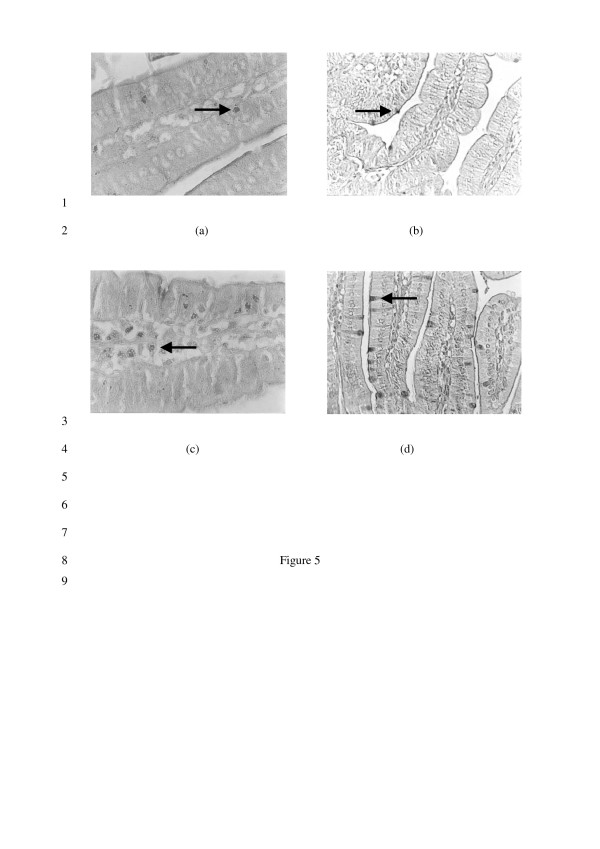
**Effect of FMSpH6 administration on mast and goblet cells expression**. Mast (a) and goblet (b) cells in the small intestine lamina propria of PBS control mice. Mast (c) and goblet (d) cells in animals that received FMSph6 for 2 consecutive days. Magnification 1000×.

When we analyzed the effect of FMSpH6 administration on mast (Fig. 5a and 5c) and goblet cells (Fig. 5b and 5d) in the small intestine lamina propria, we determined a significant increase on day 2 (154 ± 12 cells/10 fields for mast cells and 115 ± 15 cells/10 fields for goblet cells) that returned to control values (113 ± 19 cells/10 fields for mast cells and 79 ± 4 cells/10 fields for goblet cells) on day 7 for both kind of cells studied.

## Discussion

*L. helveticus *has been traditionally used in the manufacture of Swiss-type cheese and in fermented milks. This species is known to have a high proteolytic activity. Many nutrients that enter the gastrointestinal tract with the diet possess multiple effects that go beyond the classic nutritional ones. In particular, bioactive peptides derived from various animal and plant proteins were reported to have antioxidative, antimicrobial, antihypertensive and immunomodulatory activity [21,22]. Other bioactive constituents present in fermented milks, for example, are glutamine – energy source for the enterocyte and important for maintaining the integrity and the function of the intestinal barrier [23] or lactulose, that derives from the heat treatment of milk [24] and that has a recognized role as prebiotic [25]. What benefits for health and nutrition do they offer as components added to our diets? It is within this context that we aimed at furthering the knowledge of the mechanisms underlying the immunoenhancing properties of the non-bacterial fraction of milk fermented by *L. helveticus *R389 reported in a previous work [14].

Intestinal Ca^2+ ^absorption is a crucial control system in the regulation of Ca^2+ ^homeostasis because it facilitates the entry of dietary Ca^2+ ^into the extracellular compartment. Ca^2+ ^is absorbed by two different mechanisms, passive (paracellular) and active (transcellular) transport. The Ca^2+^-binding protein calbinding-D9K is responsible for the intracellular diffusion of Ca^2+ ^in the enterocyte, whereas calcineurin is not involved in the active transport of Ca^2+ ^across the epithelium [26]. Calcineurin, a serine/threonine phosphatase under the control of Ca^2+^/calmodulin, is abundantly expressed in the brain and broadly distributed in nonneural tissues as well. Among its several functions in controlling intracellular Ca^2+ ^signalling, calcineurin participates in gene regulation and external signal-mediated biological responses in many organisms and cell types. Calcineurin has been shown to regulate Ca^2+ ^pumps and exchangers to maintain Ca^2+ ^homeostasis and to regulate adaptation to high salt stress [27]. In relation to the immune system, signals transmitted by the T cell receptor (TCR) activate several biochemical pathways, including one in which calcineurin is involved. The activation of this enzyme takes place only minutes after the antigen is recognized [28]. This fact is in agreement with our observation of an enhanced expression of calcineurin+ cells in the lamina propria of the small intestine of mice during the first few minutes that followed the administration of FMSpH6 (Fig. 1). When we compared the activation of calcineurin by other means (Fig. 2) such as fermented milk or the bacterium *L. helveticus *R389 itself, we observed that the fermented milk was also able to induce calcineurin activation, probably due to its high calcium content and to the presence of natural immunoregulatory peptides [4]. The highest calcineurin activation was observed with fermented milk, possibly because of the increased peptide content due to the fermentation process. An important fact is that the probiotic strain alone was unable to induce calcineurin expression (Fig. 2).

At the cellular level, transcellular Ca2+ transport proceeds via a well controlled sequence of molecular events in which calcium channels are involved. TRPV5 and TRPV6 (originally named ECaC1 and ECaC2) form a distinct subfamily within the superfamily of transient receptor potential channels (TRPs). These channels fulfill important physiological functions ranging from phototransduction, olfaction and heat and cold sensation to epithelial calcium transport [29]. TRPV5 and TRPV6 are by far the most Ca2+-selective channels of the TRP superfamily and constitute the rate-limiting influx step in active Ca2+ (re)absorption that takes place in kidney, proximal intestine (duodenum) and placenta [30]. In mouse, TRPV6 is more ubiquitously expressed than in human, whereas TRPV5 expression is mainly restricted to the kidney. TRPV6 protein expression, in the mouse and human gastrointestinal tract, is essentially restricted to the epithelial cells, and is largely confined to the apical cell surface. In duodenum, TRPV5 and TRPV6 proteins are present in a thin layer along the apical membrane of the villous tips. As this is the major site for Ca2+ absorption, TRPV5/6 expression patterns are in line with the role of these proteins in dietary Ca2+ uptake [31]. In our work, using an anti-mouse TRPV6 antibody (Fig. 3), we observed immunopositive fluorescent staining for the TRPV6 calcium channel in the brush-border membranes of duodenal absorptive epithelial cells in the villi of control animals. In mice that received the FMSpH6, the expression of TRPV6 was enhanced, leading us to conclude that the soluble factors present in the FMSpH6 increased the expression of calcium channels. It was reported that soluble factors such as Vitamin D, estrogen and dietary Ca^2+ ^positively regulate the expression of duodenal TRPV6 [31].

Cell adhesion molecules are cell surface glycoproteins thought to represent important regulators of tissue morphogenesis. Members of the cadherin protein family are cell adhesion molecules requiring Ca^2+ ^for binding activity. Originally, four cadherins were described: epithelial (E-), neural (N-), placental (P-) cadherins and L-CAM [32]. Although the intestine expresses several members of the cadherin family, E-cadherin is the predominant cell adhesion molecule in the intestinal epithelium [33]. In our study, the expression of E-cadherin (pictures not shown) in the brush border of villi was not modified after FMSpH6 administration, which would indicate that the active components of the FMSpH6 act at different and specific levels, thus underlying the importance of the knowledge not only of the active constituents responsible for each effect but also of the target sites.

In higher animals, calcineurin is known to regulate the transcription of the T-cell growth factor, interleukin-2. Dephosphorylation of the transcriptional factor NF-ATp (nuclear factor-activated T cells) by calcineurin is required for the translocation of NF-AT from the cytoplasm to the nucleus in response to an increased intracellular Ca^2+ ^level [27]. Lamina propria T cells differ from peripherial blood T cells in the fact that the former are better activated by CD2 and CD28 stimulation and show a high expression of IL-2 and IFNγ [34]. We observed that the oral administration of FMSpH6 increased the number of IL-2+ and TNFα+ cells (Fig. 4). Since the calcineurin pathway is part of intracellular signals at different levels [35], we hypothesized that the calcium content of milk induced the activation of calcineurin. The enhanced content of peptide fractions in the FMSpH6 compared to milk efficiently would have signaled cytokine production by the T cell population, in agreement with the enhanced number of CD4+ T lymphocytes induced by FMSpH6. The results obtained in this work indicate that the immune cells involved in the adaptative immunity, as the CD4 T population, were also activated by the components of the FMSpH6. However, we cannot rule out the possibility that calcineurin activation may have simultaneously occurred in macrophages, since the presence of the Ca2+/calmodulin-dependent protein phosphatase calcineurin was also reported in macrophages [36]. It was demonstrated that lamina propria B cells play a significant role in regulating lamina propria T cells. Additionally, intestinal epithelial cells can participate in antigen presentation to T cells and the cytokines secreted by the former have direct effects on the latter [34]. In this sense, in a previous paper (14) we demonstrated that FMSpH6 did not induce S-IgA specific, thus we believe that, the activation of T cells by FMSpH6 observed in the lamina propria could follow a pathway of activation, up to day unknown for us, which differ from the classical ones through the TCR for peripherial blood T cells.

Mast cells cross the dividing line between innate and acquired immune responses because of the presence of membrane-bound, polyclonal, antigen-specific IgE, which mediates degranulation via antigen recognition [37]. Over the past few years a great deal has been learned about the potential of mast cells as effector cells in a wide range of immune and inflammatory responses. These cells have an important role in allergic responses through IgE-mediated cell activation. However, the non-IgE-mediated activation pathways of mast cells, which may induce the production of selected mediators, including cytokines [38], are probably of more relevance to both host defense and chronic inflammatory disorders. In our work, an increase in the number of mast cells in the lamina propria was observed after FMSpH6 administration (Fig. 5c). It was reported that, in certain cases, mast cells produced cytokines in response to a stimulus that did not induce degranulation [38]. There is growing evidence that mast cells recognize and react, often in a beneficial manner, to a wide range of microorganisms and their products [39], favouring their clearance, which could also be our case. Edwards *et al*. [40] reported that diets containing different protein sources were able to increase the number of colonic mast cells. The return of the number of mast cells to control values on day 7 could be due to the increase in the number of IL-10 cells, as reported in a previous work [14] since IL-10 is a very potent inhibitor of mast cells, probably because of the need to maintain intestinal homeostasis. Goblet cells, present in both the small and large intestine, release mucus granules from the apical cytoplasm after an inflammatory stimulus. Mucus secretion is triggered by both direct stimulation by immune complexes and chemical agents and indirect stimulation by mediators released by histamine and lymphokines [41]. However, mucus secretion is also stimulated by other factors such as neurogenic factors and resident microflora [42]. In a previous work in our laboratory, Gauffin Cano *et al*. [43] reported the increase in the number of goblet cells after the administration of the probiotic strain *L. casei *CRL 431 in a malnourished mouse model. Goblet cells could be considered as a possible indication of an increased non-specific barrier or an increase of the innate response. In our model of healthy animals, the proliferation of goblet cells (Fig. 5d) cannot be related to an inflammatory process, as indicated by the results obtained with the haematoxilin-eosin study (pictures not shown). Breves *et al*. [44] reported that the oral administration of oligosaccharides modified the functional parameters of the intestinal tract of pigs, increasing the number of ileal goblet cells.

In conclusion, in the present work, we demonstrated the significant contribution of the oral administration of the fermented milk supernatant from milk fermented by *L. helveticus *R389 for the improvement of the immune and physiological parameters of the gut. The enhanced expression of TRPV6 channels in the duodenum indicates an improved capacity for dietary Ca^2+ ^uptake. An enhanced expression of calcineurin able to activate the transcriptional factor NFAT to regulate IL-2 and TNFα production and the increase in local mast and goblet cells would point to an improved state of mucosal surveillance at sites of infection. The oral administration of FMSpH6 transiently affected the gut mucosal immunity and gut functioning. The induction of transient effects would assure temporal effects that would not persist longer than the feeding period. The transient activation of calcineurin and calcium transporters by the FMSpH6 would enhance gut mucosal innate immunity and calcium availability, reinforcing the innate defence mechanisms and the nutritional status of the host. The enhanced presence of goblet cells would insure an improved synthesis of mucus, which has important immune and non-immune functions that would help to protect the epithelial surface. All these events improve the intestinal barrier and functioning, increasing host protection against infections, as we recently demonstrated [15].

## Conclusion

The oral administration of the FMS from fermented milks may positively affect the milieu of the intestinal lumen. We demonstrated that soluble dietary constituents present in the lumen can influence the functions of the intestine and increasing immunological parameters without altering intestinal homeostasis, thereby enhancing the health condition of the host.

## Abbreviations

FMSpH6 = fermented milk supernatant from milk fermented by *L. helveticus *R389 (at pH 6-controlled).

## Competing interests

The author(s) declare that they have no competing interests.

## Authors' contributions

GV contributed in designing the study, performed the experimental assays and analysis, data analysis, prepared and wrote the manuscript and contributed to the discussion. CM provided the strain used in this study and discussed the manuscript, GP conceived the study and contributed to its design, coordination, supervision and to the manuscript discussion and conclusions. All the authors read and approved the final manuscript.
